# Killian’s is it a True Dehiscence? An Anatomical Perspective

**DOI:** 10.7759/cureus.10420

**Published:** 2020-09-13

**Authors:** Shivesh Maharaj, Nicolas Fitchat

**Affiliations:** 1 Otorhinolaryngology, Charlotte Maxeke Johannesburg Academic Hospital, Johannesburg, ZAF; 2 Otolaryngology - Head and Neck Surgery, Charlotte Maxeke Johannesburg Academic Hospital, Johannesburg, ZAF

**Keywords:** killan’s triangle, zenker’s diverticulum, posterior pharyngeal wall

## Abstract

Objective

Zenker’s diverticulum is a pulsion outpouching from the posterior pharyngeal wall. The anatomy of the wall has been proposed to be dehiscent in the region of the Killian’s triangle, between the thyropharyngeus muscle (inferior pharyngeal constrictor) and the cricopharyngeus muscle. A dehiscence is a bursting open, splitting or gaping along natural or sutured lines. To the best of our knowledge, there have not been any studies to histologically analyze the posterior pharyngeal wall and the exact location of the dehiscence. We thus aim to determine the presence and characteristics of this area of possible dehiscence.

Methods

Fifty-eight cadavers were analysed. A portion of tissue was excised within the borders of the Killian’s triangle, being the inferior border of the oblique inferior constrictor muscle and the superior border of the cricopharyngeus muscle from the posterior wall of the pharynx. Four longitudinal sections were sampled from each cadaver including: left lateral, left medial, right medial and finally the right lateral aspect of the posterior pharyngeal wall. These samples were then embedded in wax and cut with a microtome at 5 microns. They were then placed on microscope slides and stained with Haematoxylin and Eosin and analysed in terms of thickness and histology.

Results

There was significant overlapping of the thyropharyngeus and cricopharyngeus muscles seen macroscopically in all cadavers that were dissected. No obvious area of dehiscence was found in any of the specimens, however, there were variations in the thickness of the posterior pharyngeal wall within the thyropharyngeus muscle. When comparing the left- and the right-hand sides of the thyropharyngeus, the mean measurement of the left medial muscle sample was found to be significantly thinner than the mean measurement of the right medial muscle sample (95% CI Inf to -44.39, p-value = 0.0189). The average of both the thickest and thinnest muscle measurements for each of the four samples was then compared. The average left medial muscle layer was found to be significantly thinner than the average right medial muscle layer (95% CI Inf to -9.81, p-value = 0.03822).

Conclusion

This study demonstrated that the left thyropharyngeal muscle was thinner than the right. However, no dehiscent areas were found in any of the specimens. Significant overlapping of the cricopharyngeus and thyropharyngeus muscles was noted. Thus, we propose that the hypopharyngeal pouch, given enough intraluminal pharyngeal pressure, may occur between the fibres of the inferior pharyngeal constrictor muscle rather than between the cricopharyngeus and the inferior pharyngeal constrictor muscles. As a dehiscence occurs between a natural or sutured line, of which there is neither in the thyropharyngeus muscle, we propose that the term Killian’s dehiscence is a misnomer and that the defect instead meets the definition of a hernia.

## Introduction

Zenker’s diverticulum is an outpouching of the mucosa and submucosa located in the posterior hypopharyngeal wall [1,2]. The exact aetiology and pathophysiology of the hypopharyngeal pouch have not been established, however, various theories have been postulated. This dehiscence was first discovered in 1769 by Ludlow who identified it as an abnormal dilatation in the posterior pharyngeal wall during the post-mortem examination of a patient who, during life, complained of dysphagia [3]. 

The area that has been postulated to be the site is known as Killian’s dehiscence as described by Killian in 1908 [1]. The dehiscent area of muscular weakness is a triangular region formed between the two pharyngeal and oesophagal muscles, the inferior pharyngeal constrictor and the cricopharyngeus. The medical definition of a dehiscence is a bursting open, splitting, or gaping along natural or sutured lines [3].

Killian stated that the triangle is always present yet some authors argue that there is considerable overlap of the inferior constrictor muscular fibres and thus have cast doubt regarding the presence and prevalence of the dehiscence [1,2].

Cadaver studies have demonstrated the replacement of normal muscle and connective tissue with fibro adipose tissue [4,5]. This further contributes to the increased intraluminal pressure due to stiffness and decreased compliance of the muscle.

To the best of our knowledge, there have not been any studies to histologically analyse the posterior pharyngeal wall to explore the exact location of the Killian’s dehiscence. We thus aim to determine the possible location for the development of a Killian’s Dehiscence.

## Materials and methods

Fifty-eight cadavers (28 males and 30 females) were dissected in the study after approval from the University of the Witwatersrand Human Research Ethics Committee number W-CJ-140604-1. A portion of tissue was excised from the posterior pharyngeal wall and from the inferior border of the oblique inferior constrictor muscle till the superior border of the cricopharyngeus muscle.

**Figure 1:**
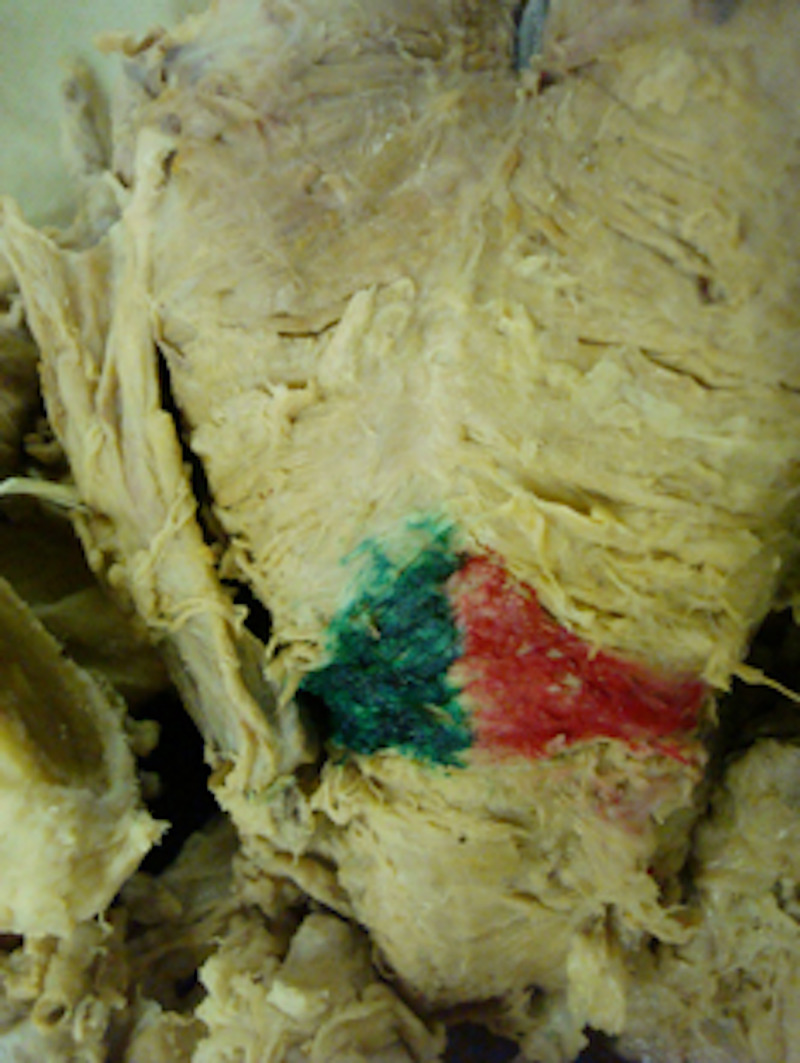
A macroscopic view of the posterior pharynx in a cadaveric specimen demonstrating the inking of the outer adventitial layer of the Killian’s Triangle. Left was inked green and right was inked red.

**Figure 2:**
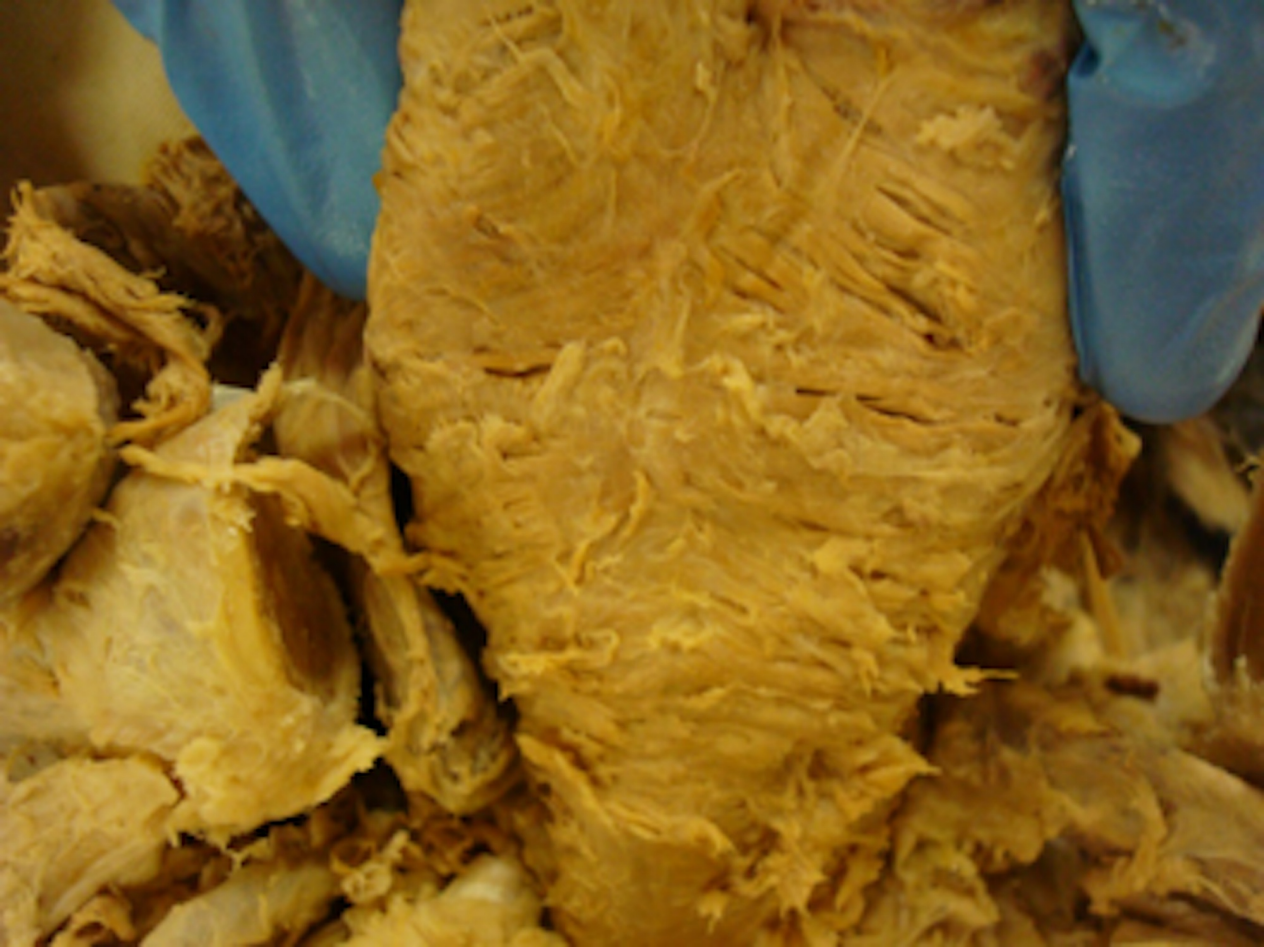
A macroscopic view of the posterior pharynx in a cadaveric specimen demonstrating continuous muscular overlap between the thyropharyngeus and cricopharyngeus muscles. No obvious dehiscence visualised.

Four longitudinal sections were sampled from each cadaver, including left lateral, left medial, right medial and finally the right lateral aspect of the posterior pharyngeal wall. The adventitial layer of the pharynx was inked with red or green in order to identify the left and right sides of the excised portion of the tissue during later histological analysis (Figures 1 and 2). Left was inked green and right was inked red. The median fibrous raphe, to which the inferior pharyngeal constrictor is attached, was used to divide between the left and right sides. These samples were then embedded in wax and cut with a microtome at 5 microns. They were then placed on microscope slides and stained with Haematoxylin and Eosin. To compare means of the muscle thickness of four samples taken (left lateral, left medial, right medial and right lateral), parametric testing by means of a paired Student´s t-test was used.

 

## Results

The 28 male cadavers had a mean age of 81 years and a mean height of 173 cm. The 29 female cadavers had a mean age of 76 years and a mean height of 162 cm. All cadavers were Caucasian in ethnicity. No obvious area of dehiscence was found in any of the specimens; however, there were variations in the thickness of the muscle of the posterior pharyngeal wall. The exact location of Killian’s dehiscence was not identified in any of the specimens as we found the muscle layer to be continuous. When comparing the left- and the right-hand sides, the mean of the thickest measurement of the left medial muscle sample was found to be significantly thinner than the mean of the thickest measurement of the right medial muscle sample (95% CI Inf to -44.39, p-value = 0.0189).

The average of both the thickest and thinnest muscle measurements for each of the four samples was then compared. The average left medial muscle layer was found to be significantly thinner than the average right medial muscle layer (95% CI Inf to -9.81, p-value = 0.048). Therefore, the muscular layer in the left medial aspect of the posterior pharyngeal was significantly thinner than the right medial muscular layer.

**Figure 3:**
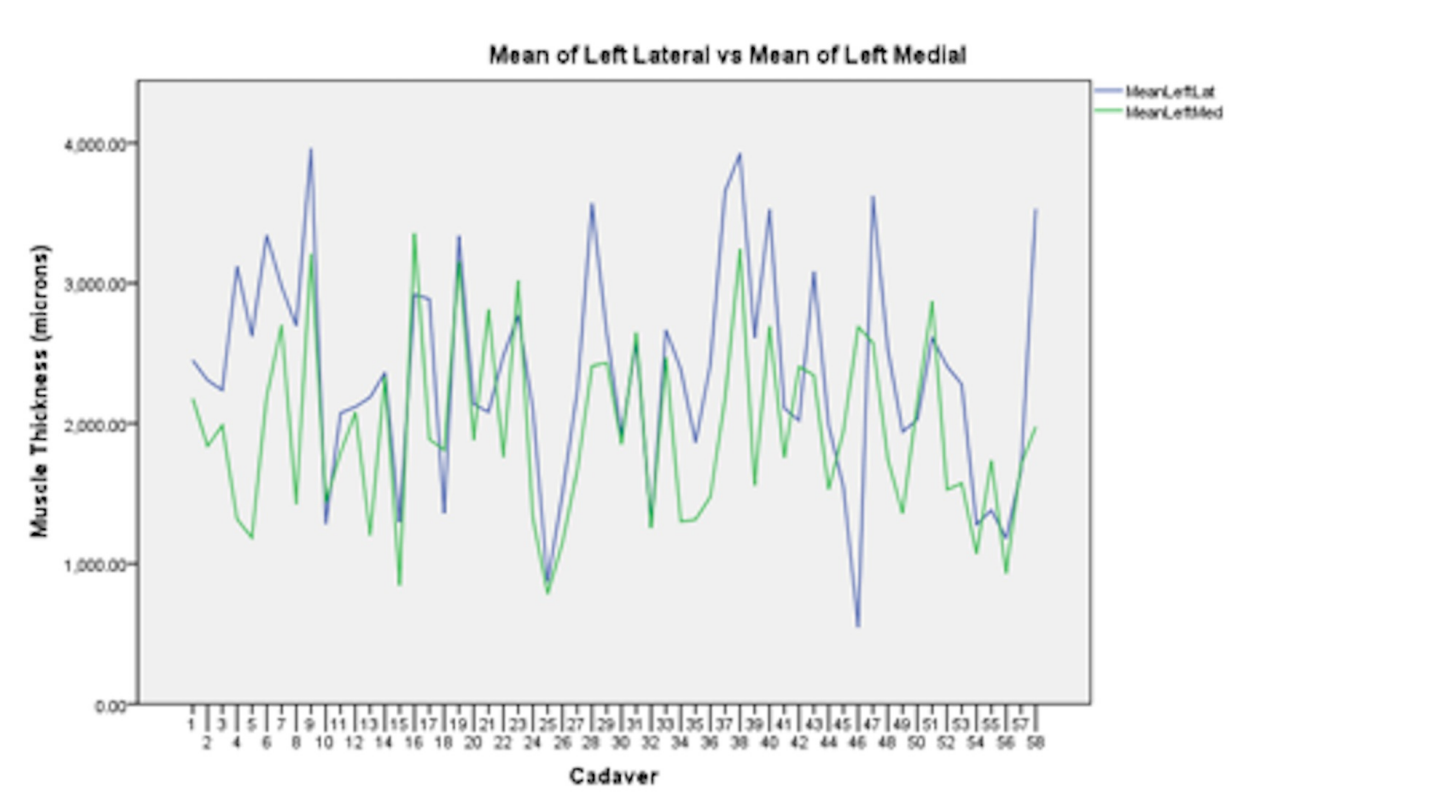
A graph comparing the muscle thickness (microns) of the left medial muscle layer (green line) versus its corresponding left lateral muscle layer (blue line).

The left lateral thickest muscle measurement was found to be significantly thicker than its corresponding left medial thickest muscle measurement (95% CI 184.75 to Inf, p-value = 0.0003). The left lateral thinnest muscle measurement was found to be significantly thicker than its corresponding left medial thinnest muscle measurement (95% CI 251.15 to Inf, p-value = 0.00014; Figure 3).

**Figure 4:**
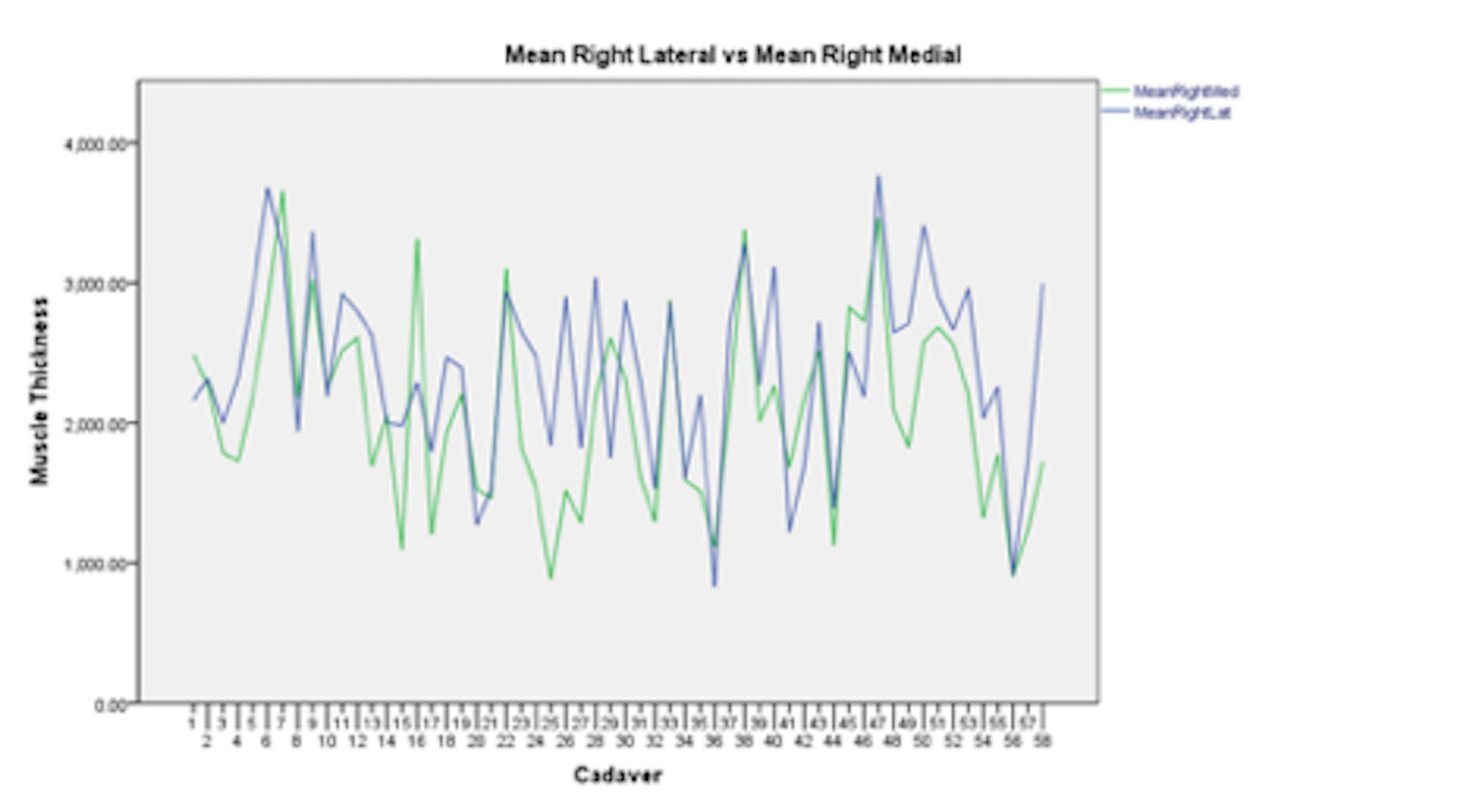
A graph comparing the muscle thickness (microns) of the right medial muscle layer (green line) versus its corresponding right lateral muscle layer (blue line).

The right lateral thickest muscle measurement was found to be significantly thicker than its corresponding right medial thickest muscle measurement (95% CI 69.72 to Inf, p-value = 0.0098; Figure 4). The right lateral thinnest muscle measurement was found to be significantly thicker than its corresponding right medial thinnest muscle measurement (95% CI 247.01 to Inf, p-value = 0.0000019).

**Figure 5:**
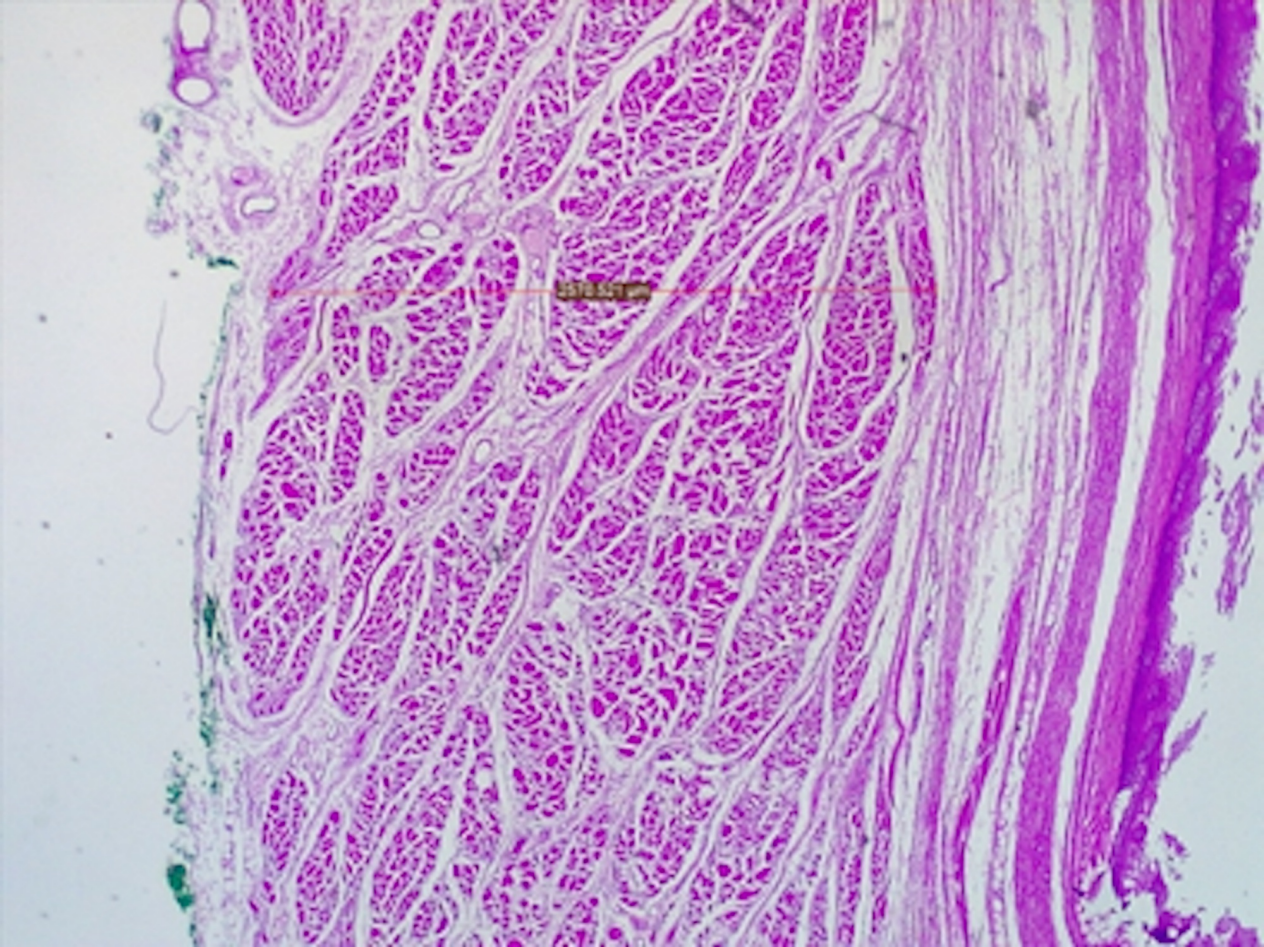
A microscopic view of the excised portion posterior pharynx within the borders of the Killian’s triangle showing the adventitial layer inked (inked green as from the left side), a continuous muscle layer and an inner mucosal layer.

The average between the thicker and thinner muscle measurements of the four samples was also compared. The average left lateral muscle sample was significantly thicker than the average left medial muscle sample (95% CI 269.17 to Inf, p-value = 0.0000072) and the average right lateral muscle sample was significantly thicker than the average right medial muscle sample (95% CI 185.92 to Inf, p-value 0.000028). These measurements may be appreciated in Figure 5 that shows the stained excised portion of the posterior pharyngeal wall.

## Discussion

The pharyngeal pouch has been described over 150 years ago and was postulated to occur in a dehiscent area known as the Killian’s dehiscence as described by Killian [1]. The medical definition of a dehiscence is a bursting open, splitting, or gaping along natural or sutured lines. Various authors have claimed that such a weak spot does not truly exist as there was an overlap of the lower pharyngeal muscles that re-inforced the region.

Kelly and Kuncl dissected 21 non-fixed specimens and only reported the triangle in six of those cadavers. They, however, did not strictly define the boundaries of the triangle. In our study of 58 specimens, no obvious area of dehiscence was identified during the time of cadaveric dissection [6]. Furthermore, there was no dehiscent area found during the macroscopic or microscopic assessment of the regions, nor was there a hypopharyngeal pouch identified in any of the cadaveric specimens. Instead, a thinner wall was identified on the left side of the hypopharynx.

One of the challenges that have previously been encountered was the variation described in the pattern of the hypopharyngeal muscles. We found the muscle fibres to be flat and thick in the overlapping regions, which was in keeping with studies done by Anagiotos. They described the existence of a triangle in 9 out of 15 males and 11 out of 32 females. However, none of their specimens had a pharyngeal pouch [7]. 

There have been many theories as to why the pharyngeal pouch has a left-sided predominance. Killian attributed this due to the muscle wall weakness on the left side; this is in keeping with our findings, as we found a thinner left lateral wall in our study.

The pharyngeal phase of swallowing depends on a pressure gradient from the oropharynx to the hypopharynx. During swallowing, a negative pressure is generated, at the junction of the hypopharynx and oesophagus, by elevation of the larynx. Coupled with this is the cricopharyngeal relaxation that allows the bolus to pass to the oesophagus [8,9]. Cross et al. used manometry to demonstrate that a raised intra-luminal pressure results in an outpouching of the mucosa [10]. The raised intraluminal pressure may be due to the spasm of the pharyngo-oesophageal segment and dysfunction of the cricopharyngeal muscle [10-13].

Frieling et al. described an abnormal sequence of pharyngeal and oesophageal contractions [14]. Barthelen et al. proposed that there was an anatomical basis for the disease as it was due to patients having a large constrictor-cricopharyngeus space and that there was mucosal prolapse due to loss of tissue elasticity with age [15].

Some authors have suggested a primary myopathy of the cricopharyngeus muscle (Table 1). There may even be a defect resulting in the degenerative muscle fibres being replaced by fibro adipose tissue [16-20]. This was not found in our study.

**Table 1:** A table with the characteristics of the posterior pharynx as described by previous studies.

Study	Authors	Number of Specimens	Results	Conclusions	Year Published
Diseases of the mouth and eating	Killian [1]	1			1908
Myology of the pharyngoesophageal segment: gross anatomic and histologic characteristics	Kelly and Kuncl [6]	21	4/14 males 2/7 females		1996
Anatomical aspects of hypopharyngeal diverticula	Perrot [20]	40		Weak place - no triangle identified	1961
Morphometric and anthropometric analysis of Killian’s triangle	Anagiotos [7]	47	9/15 males 11/32 females	Triangle found, but no dehiscence	2010

A hernia is a term used to describe a bulge or protrusion of an organ through the structure or muscle that usually contains it. To date, the exact pathogenesis of the pharyngeal pouch has still not been fully understood and rather been considered as a field of ongoing work. This study demonstrates that the left posterior pharyngeal wall muscle is thinner than the right. However, no dehiscent areas were found in any of the cadaveric specimens that were looked at, but rather the presence of considerable overlap between the thyropharyngeus and cricopharyngeus muscles. 

Thus, we propose that the term “Killian’s dehiscence” is a misnomer as there is no true anatomical dehiscence as there is no splitting along a natural line within the muscle. However, the area of weakness as a result of a thinner muscle layer may predispose to the formation of the pharyngeal diverticulum as a herniation through the muscle of the inferior pharyngeal constrictor given enough pharyngeal intra-luminal pressure. We propose the term: Maharaj-Fitchat hernia. 

## Conclusions

Zenker’s diverticulum is a pulsion outpouching from the posterior pharyngeal wall. The anatomy of the wall has been proposed to be dehiscent in the region of the Killian’s triangle. In our study, no obvious area of dehiscence was found in any of the specimens, however, there were variations in the thickness of the posterior pharyngeal wall. The exact location of Killian’s dehiscence was not identified on any of the specimens. 

When comparing the left- and the right-hand sides, the mean of the thickest measurement of the left medial muscle sample was found to be significantly thinner than the mean of the thickest measurement of the right medial muscle sample. This study demonstrated that the left posterior pharyngeal wall was thinner than the right. However, no dehiscent areas were found on any of the specimens.
